# Molecular characterization of human isolates of *Strongyloides stercoralis* and *Rhabditis* spp. based on mitochondrial cytochrome c oxidase subunit 1 (*cox1*)

**DOI:** 10.1186/s12879-019-4407-3

**Published:** 2019-09-05

**Authors:** Mandana Fadaei Tehrani, Meysam Sharifdini, Farzaneh Zahabiun, Robabeh Latifi, Eshrat Beigom Kia

**Affiliations:** 1 Department of Medical Parasitology and Mycology, School of Public Health, Tehran University of Medical Sciences, Tehran, Iran; 2 Department of Medical Parasitology and Mycology, School of Medicine, Guilan University of Medical Sciences, Rasht, Iran

**Keywords:** *Strongyloides stercoralis*, *Rhabditis* spp., PCR, *cox1*

## Abstract

**Background:**

Due to the similarity of *Strongyloides stercoralis* with free-living nematodes of *Rhabditis* species they might be miss-diagnosed with each other in microscopical examination of stool samples. The aim of this study was molecular characterization and differentiation of human derived isolates of *S. stercoralis* and *Rhabditis* species based on the mitochondrial gene of cytochrome c oxidase subunit 1 (*cox1*) amplification.

**Methods:**

Using parasitological methods, ten isolates of *S. stercoralis* and three isolates of *Rhabditis* spp. were obtained from fresh stool samples of patients and the genomic DNA of the samples were extracted. PCR amplification of *cox1* gene was carried out for all the isolates and the products were sequenced.

**Results:**

The phylogenetic analysis illustrated that *S. stercoralis* and *Rhabditis* spp. isolates were placed in two distinguishable separate clades. Inter-species genetic variation between isolates of *S. stercoralis* and *Rhabditis* spp*.* were ranged from 13.5 to 14.5%.

**Conclusions:**

*Cox1* gene was a suitable marker for discrimination of *S. stercoralis* from *Rhabditis* spp. retrieved from human in the current study. The availability of gene sequence information will be helpful in the future development and validation of discriminatory PCR-based assays of these nematodes.

## Background

In genus *Strongyloides*, two species are known that infect human beings: *Strongyloides fuelleborni* that has local importance among human soil transmitted helminthes [1], and the soil-transmitted threadworm, *Strongyloides stercoralis*, which is one of the most neglected among neglected tropical diseases [2]. It is estimated that 30 to 100 million people are infected with *S. stercoralis* worldwide [1], mainly in tropical and subtropical areas. However, information on infection rates is missing in many countries [2]. In immunocompetent individuals, *S. stercoralis* commonly causes chronic, asymptomatic infection but a change in immune status can lead to an increase in parasite burden, hyperinfection syndrome, dissemination [3] and even death if not treated properly [4, 5]. Corticosteroids have a particularly strong and specific association with the development of such infections; other immunosuppressive therapies and underlying conditions may also predispose to dissemination [3]. This imposes a serious threat to immunocompromised patients. Thus, an early laboratory diagnosis of this infection has special clinical importance.

Given the needs for the application of highly sensitive and specific methods for early diagnosis of strongyloidiasis, various parasitological, immunodiagnostic and molecular assays have been evaluated [6] and novel tests have been implemented [7]. However, a reliable gold standard for the diagnosis of this unique nematode is still lacking [6, 7]. Although molecular biology technique of polymerase chain reaction (PCR), that can detect DNA of the parasite, is highly specific, it should not yet be recommended for universal screening, nor as a standalone method for individual diagnosis of strongyloidiasis; and it has rather a role as a confirmatory test [7]. In the other words, in clinical parasitology, molecular diagnosis could be extremely useful when a specific parasite or group of parasites are to be studied, however they cannot yet replace microscopy as routine diagnostic tests [8]. In fact, routine microscopy is a catchall technique whereby the presence of any parasite in the clinical specimen can be detected [8].

There are many parasitological tests available for detection of *S. stercoralis* larvae in stool samples [6]. Agar plate culture (APC) is more efficient for detection of *S. stercoralis* in comparison with other parasitological tests including direct smear preparation and formalin-ether concentration [9]. Despite the efficiency of this culture method, it raises a possibility for the growth of other nematodes including hookworms [9], *Trichostrongylus* and free-living nematodes of the genus *Rhabditis* (Rhabditida) species [10]*.* In infectivity with the major soil transmitted nematodes of human, including *Ascaris lumbricoides*, *Trichuris trichiura*, and hookworms (*Ancylostoma duodenale* and *Necator americanus*) [1], the main diagnostic stage in stool sample is eggs [11]. While, Rhabditoid nematodes have been reported releasing larvae in human [12–15]. They are not among main helminthes of human [1], and there is no estimation on population infected in endemic areas of strongyloidiasis in the world. However, due to the similarity with *S. stercoralis*, in terms of the presence of larval stage in stool samples [12–15], their morphological differentiation is a challenge, requiring a well-trained microscopist.

Human infections with *Rhabditis* spp. nematodes have been reported as scattered sporadic reports from different parts of the world, for example from China [14], Korea [15], Japan [16], Brazil [13], Rhodesia [17], Germany [18] and Iran [12]. In the past, morphological criteria were used for discrimination of *Rhabditis* species from *S. stercoralis* [12–17, 19]. However, given the increasing numbers of immunocompromised individuals throughout the world [20] and necessity for accurate and early detection of *S. stercoralis*, a more reliable method of differentiation is needed. Utilization of sensitive and specific diagnostic approach of molecular-based methods, associated with sequencing, will be helpful in this aspect. However, such utility is currently restricted due to lack of accurate gene sequence information of pathogenic species of *Rhabditis*. In this view, in order to pave the way for future development of an discriminative DNA based assay to be used in clinical setting, present study aimed to differentiate human derived isolates of *S. stercoralis* and *Rhabditis* species using molecular characterization of mitochondrial gene of cytochrome c oxidase subunit 1 (*cox1*) amplification.

## Methods

### Sample collection

This study was performed in 2016–2017. In order to obtain human isolates of *S. stercoralis* and *Rhabditis* spp., fresh stool samples were collected from patients who referred to the Diagnostic Laboratory of Strongyloidiasis in School of Public Health, Tehran University of Medical Sciences, and residents in endemic areas of strongyloidiasis in Iran [21] including Mazandaran, Guilan and Khuzestan Provinces.

### Parasitological methods

Collected stool samples were examined by parasitological methods including direct smear preparation, formalin-ether concentration and nutrient agar plate culture (APC). For APC roughly 3 g of each stool sample was subjected to nutrient agar media [9]. Then, after 48–72 h incubation in room temperature at about 28 °C–30 °C, the plates were examined under a stereomicroscope for the presence of any larvae and free-living adults of *S. stercoralis* and *Rhabditis* species. In case of infectivity with *Rhabditis* spp., triple stool samples were taken to ensure true parasitic infection; and the patients were considered infected if all their triple stool samples had been found positive for *Rhabditis* species.

To prepare the infected samples for further molecular analysis, surface of nutrient agar plates were washed out with lukewarm phosphate buffer saline and after collection of larvae [10] they were preserved in 75% ethanol. Additionally, infected stool samples were also preserved in 75% ethanol after washing by sterile distilled water and sedimentation by centrifuge.

### DNA extraction

Ethanol preserved cultivated and harvested larvae, and stool sediments containing larvae were washed three times with sterile distilled water by centrifugation at 5000×g for 5 min to remove ethanol. Then, they were subjected to five cycles of freezing in liquid nitrogen and thawing in boiling water. Following this, approximately 300 mg glass beads (0.5 mm in diameter) were added and shaken intensively for 5 min. Subsequently, the genomic DNA was extracted by genomic DNA extraction kit (GeneAll Exgene, Seoul, Korea) according to the manufacturer’s instructions and stored at − 20 °C until the performance of PCR amplification.

### PCR amplifications and sequencing

Polymerase chain reactions (PCR) based on the *cox*1 gene were performed with primers cox F (5′TGG TTT GGG TAC TAG TTG-3′) and cox R (5′-GAT GAG CTC AAA CTA CAC A-3′) [22]. Every PCR amplification was carried out in a final reaction mixture containing 15 μL of PCR mix including 1.25 U Taq DNA polymerase, 200 μM of dNTPs and 1.5 mM MgCl2 2x red PCR Master Mix (Ampliqon, Odense, Denmark), 20 pmol of each primer and 2 μL of each sample DNA. A negative control (distilled water) and a positive control (extracted DNA from *S. stercoralis* filariform larva) were applied in each run. The thermal PCR profile (Applied Biosystems 2720 Thermal Cycler, California, USA), included an initial denaturation step at 95 °C for 6 min, followed by 35 cycles of denaturation at 95 °C for 45 s, annealing at 55 °C for 60 s and extension at 72 °C for 60 s, followed by a final extension step at 72 °C for 6 min.

The amplified products were run on a 1.5% agarose gel and visualized using a UV transilluminator.

These PCR products were submitted to Bioneer Company (South Korea) and were sequenced with Sanger method in both directions using the same PCR primers. All obtained sequences were edited and trimmed by Chromas software version 2.6.1 (South Brisbane, Australia).

Analysis of sequencing data was carried out using the National Center for Biotechnology Information BLAST programs and databases (http://www.ncbi.nlm.nih.gov/). Multiple sequence alignments were performed with Clustal W method using Bioedit software version 7.1 (http://www.mbio.ncsu.edu/bioedit/bioedit.html) and compared with the sequences present in GenBank database.

### Phylogenetic analysis

The phylogenetic tree was constructed using Maximum-Likelihood algorithm and Tamura-3-Parameter option by Molecular Evolutionary Genetics Analysis software version 6.0 (MEGA). Bootstrap with 1000 replications was utilized for determining the topology reliability of the tree.

## Results

In this study, overall ten isolates of *S. stercoralis* and three isolates of *Rhabditis* spp. were obtained from human stool samples. These isolates are listed in Table 1 to show the original province of each patient and the corresponded accession number submitted in GenBank.

**Table 1:** List of *Rhabditis* spp. (R1, R2, and R3) and *Strongyloides stercoralis* (S1 to S10) isolates with original province of patients and related accession numbers in GenBank based on amplification of *cox1* gene

Isolate	Patient original province	Accession number in GenBank
R1	Tehran	MG251327
R2	Mazandaran	MG251328
R3	Mazandaran	MG251329
S1	Guilan	MG251318
S2	Mazandaran	MG251319
S3	Mazandaran	MG251320
S4	Guilan	MG251321
S5	Khouzestan	MG251322
S6	Guilan	MG251323
S7	Khouzestan	MG251324
S8	Tehran	MG251326
S9	Mazandaran	MG251325
S10	Guilan	MG251317

**Fig. 1:**
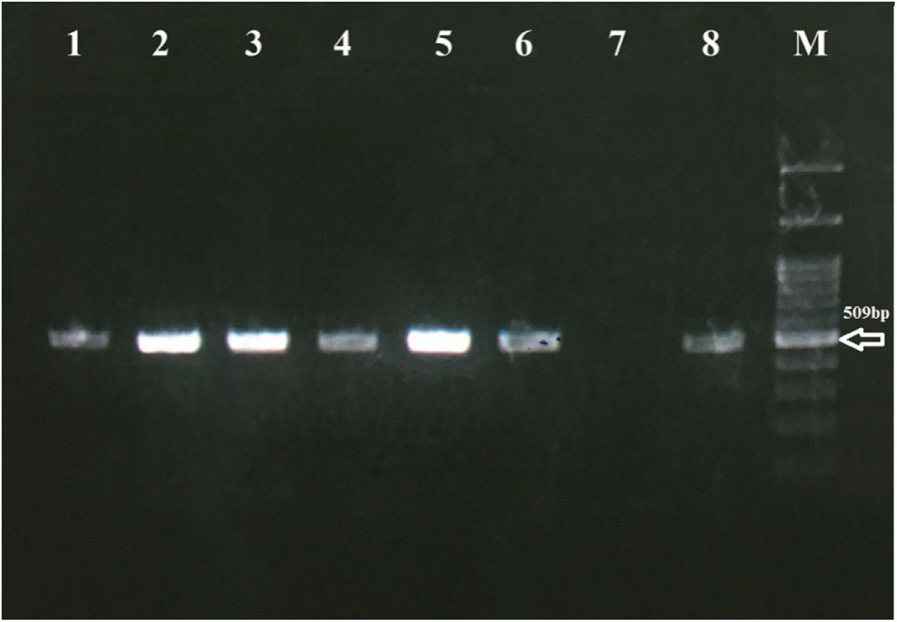
Agarose-gel electrophoresis of polymerase chain reaction (PCR) products amplified with genomic DNA from *Strongyloides stercoralis* and *Rhabditis* spp. samples. Lanes 1, 2 and 3: *S. stercoralis* samples; Lanes 4, 5 and 6: *Rhabditis* spp. samples; Lane 7: Negative control; Lane 8: Positive control (*S. stercoralis* filariform larva); and Lane M: 100-bp DNA ladder

After optimizing PCR conditions in terms of materials and thermal PCR protocol and the number of cycles, all *S. stercoralis* and *Rhabditis* spp*.* isolates were successfully demonstrated the amplification of about 509-bp target band for the *cox1* (Fig. 1). The tests were reliable when the negative control did not yield an amplification band and the positive control replicated the desired amplification band without presenting smears.

**Fig. 2:**
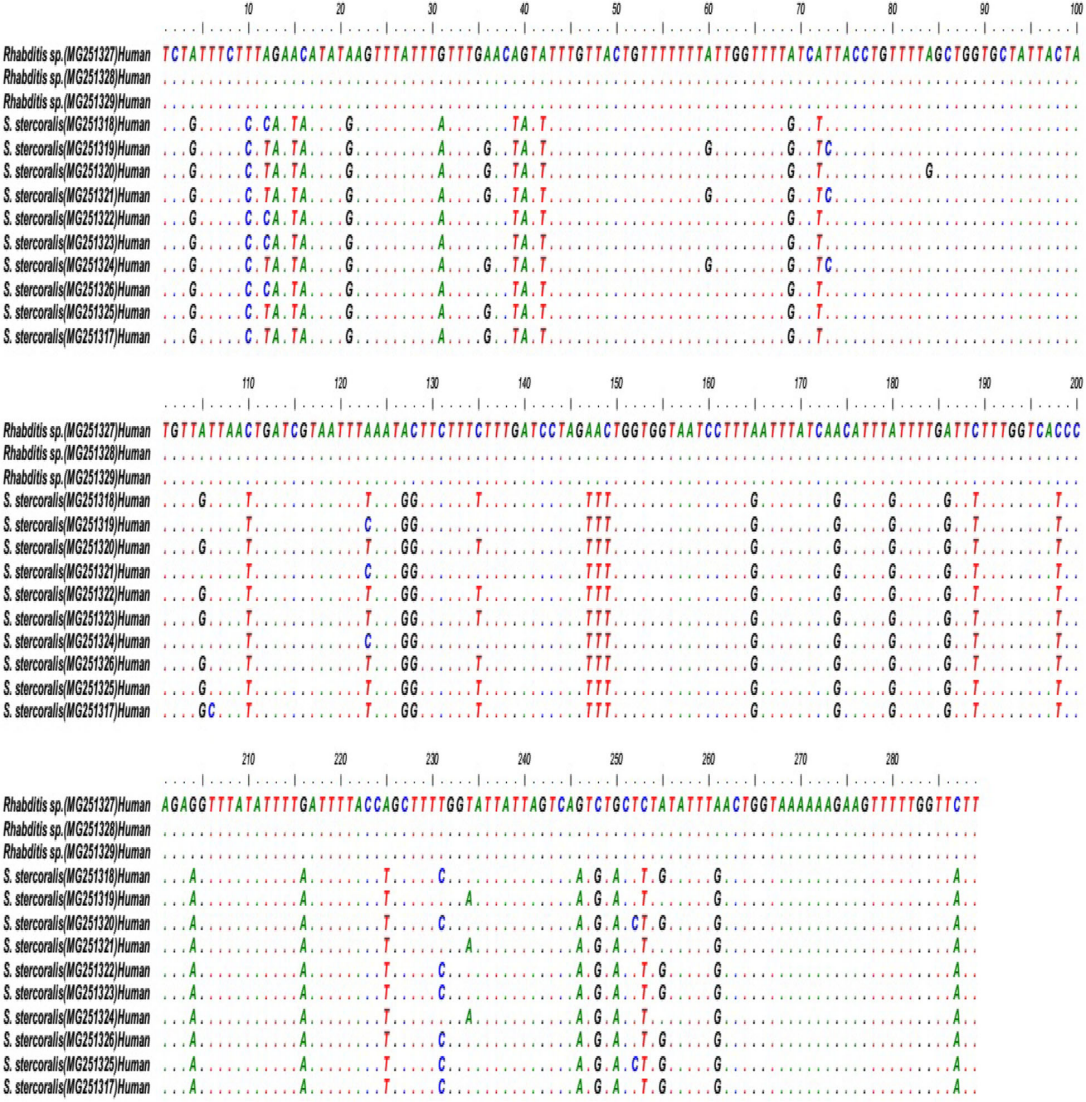
Sequence alignment of *Strongyloides stercoralis* and *Rhabditis* spp. isolates obtained in the current study based on *cox1* gene by Clustal W method via Bioedit software version 7.1 (http://www.mbio.ncsu.edu/bioedit/bioedit.html)

After trimming the sequences, the isolates were aligned and compared with the sequences available in GenBank. The sequence alignment indicated that there is high level of sequence difference between isolates of *S. stercoralis* and *Rhabditis* spp*.* (Fig. 2).

Inter-species and intra-species genetic variation between present isolates of *S. stercoralis* and *Rhabditis* spp*.* were calculated. The inter-species genetic variation between *S. stercoralis* and *Rhabditis* spp*.* were ranged from 13.5 to 14.5%. Pairwise distance indicated the presence of intra-species genetic variation of 0–3.5% among the current isolates of *S. stercoralis*, and 0 to 5.2% of those with the sequences previously available in GenBank. Furthermore, multiple alignment represented that intra-species similarity among *Rhabditis* spp. isolates was 100%.

**Fig. 3:**
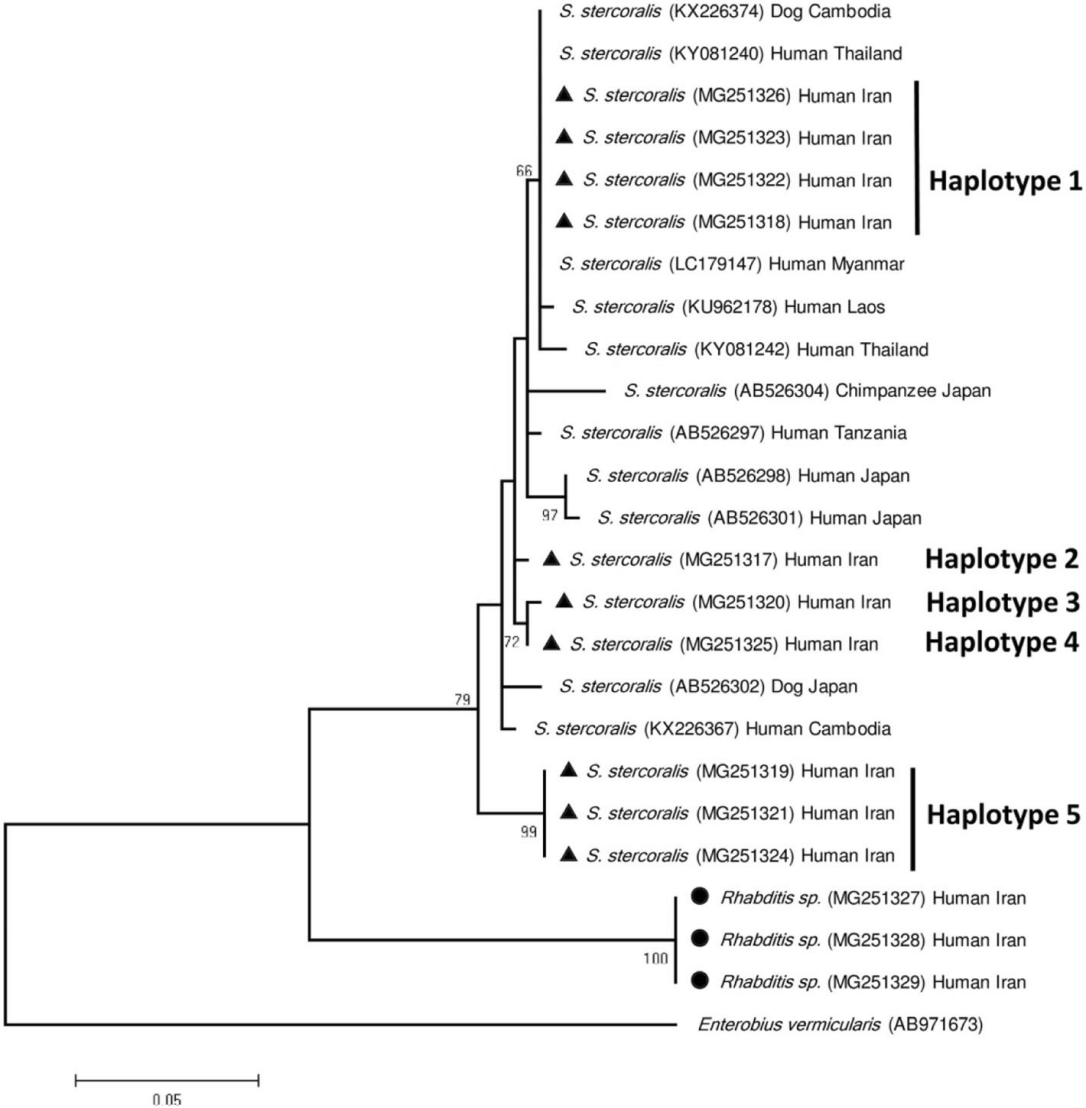
Phylogenetic tree of *Strongyloides stercoralis* (▲) and *Rhabditis* spp. (●) isolates obtained in this study and reference sequences retrieved from GenBank based on *cox1* gene sequences and constructed tree using Tamura 3-parameter model by MEGA software version 6.0. *Enterobius vermicularis* was selected as out-group. Scale bar represented 0.05 changes per nucleotide

The phylogenetic tree was constructed based on *cox1* to evaluate the haplotypes recovered in this study along with retrieved sequences from other regions of the world (Fig. 3). The phylogenetic analysis illustrated that *S. stercoralis* and *Rhabditis* spp. isolates were placed in two distinguishable separate clades. The tree indicated that the ten sequences of *S. stercoralis* isolates in the present study were consisted of five haplotypes, placing into two clusters. One cluster included four haplotypes whereas the other one consisted of merely one haplotype (Fig. 3). Intra-species genetic variation between the two clusters was calculated within 2.8 to 5.2%.

The *cox*1 sequences of all *S. stercoralis* and *Rhabditis* spp. were registered in GenBank database. The accession numbers of these sequences are shown in Table 1.

## Discussion

Genus *Rhabditis* have been reported to infect human digestive system [12–15], urinary system [16, 17, 19], and even outer ear canal [18]; and excreting larvae in stool and urine. As the parasitological diagnosis of *S. stercoralis* is the detection of parasite larvae in stool exams and other biological materials such as sputum, duodenal aspirates, gastric biopsies, cervical smear or CSF liquid [6], therefore, this study pointing out to the challenge for discrimination between *S. stercoralis* and *Rhabditis* species. Due to the differences in the managements of infected patients with *S. stercoralis* [11], the exact differentiations of these two parasites is necessary.

The essential of early diagnosis of strongyloidiasis, especially in immunocompromised individuals, urged the efforts for development of molecular biology technique, mostly based on PCR, during recent years. Although, such techniques are highly sensitive and specific, they are recommended as confirmatory tests [7]. Besides, such molecular techniques have not been developed for detection of *Rhabditis* spp. recovered from human stool samples, and on the other hand, identification of such rare parasites using morphological criteria requires expert microscopists that may be lacking among new generation of laboratory staff [8]. Therefore, in order to attempt for prevention of misdiagnosis between these parasites, the present study was designed to characterize human retrieved isolates of *S. stercoralis* and *Rhabditis* spp. based on the amplification of *cox1* gene. For this purpose, ten *S. stercoralis* and three *Rhabditis* spp. isolates were obtained from infected human stool samples and molecular approach was carried out by utilizing *cox1* gene.

Although studies comparing quantitative polymerase chain reaction (qPCR) with microscopy methods for soil-transmitted helminthes are imperfect, they do show a significant increase in sensitivity and specificity of qPCR compared with labor-intensive traditional microscopic techniques [23]. The *cox1* gene of mitochondrial DNA, which has been reported to mutate more rapidly than 18S rDNA, is useful sources of sequence data for study on different populations of *Strongyloides* species [24] and a useful target for molecular diagnosis of strongyloidiasis in human stool samples [22]. Presently, utilization of molecular-based methods in identification of *Rhabditis* species in human is very rare in clinical settings [18]. The sequence alignments of this study illustrated a considerable difference between nucleotide sequences of *S. stercoralis* and *Rhabditis* species. The inter-species genetic variation between *S. stercoralis* and *Rhabditis* spp. was 13.5 to 14.5%. According to pairwise distance of the current study isolates, intra-species genetic variation within *S. stercoralis* nucleotide sequences was 0 to 3.5%. Pairwise difference in *cox1* gene among interbreeding strains of a nematode species was reported to be usually less than 6% and that between distinct species in a genus was more than 10% [25]. Pairwise difference in nucleotides sequence of *cox1* gene among isolates of *S. stercoralis* from humans, apes and dogs was less than 4%, regardless of the host and locality of the isolates [24]. The average pairwise distances of *cox1* nucleotide sequences were 0.036 for *S. stercoralis* isolates from human and dog collected mainly from Myanmar [26].

According to the phylogenetic analysis of current study, *S. stercoralis* isolates were located in a distinct clade from *Rhabditis* species*. Rhabditis* spp. clade was included three isolates, all merely from our study, having 100% similarity with each other. There was not any available *cox1* gene sequence of *Rhabditis* species with human origin or other pathogenic isolates to be included in the constructed tree for comparison. The phylogenetic tree indicated that the ten sequences of *S. stercoralis* consisted of five haplotypes. The haplotype one, which included four isolates, showed 100% homology with GenBank registered sequences of human isolates from Thailand (KY081240), Myanmar (LC179147), and dog isolate from Cambodia (KX226374). The haplotypes two, three and four, each including one isolate, have not been already recorded from other regions in the world. Moreover, haplotype five, locating solely in separate cluster and including three completely similar isolates, was distinctive from any other haplotypes in the tree. Despite the small number of *S. stercoralis* isolates studied presently, five different haplotypes were recovered which four of them were considered as new reports. Thus far, it seems *S. stercoralis* represents diverse haplotypes and needs to be speculated further using more isolates from different regions of the world in order to investigate its genetic variations.

Among our isolates of *S. stercoralis*, haplotype one indicated 100% homology with a dog isolate from Cambodia (KX226374), belonging to the population indistinguishable from the population of *S. stercoralis* isolated from humans in Cambodia [27]. In mentioned study, two genetically different populations of *Strongyloides* spp. were found in dogs, one of which that the majority of the worms belonged, appeared to be restricted to dogs; the other population was shared with humans [27]. In another study, using nuclear and mitochondrial markers, phylogenetic relationships among *S. stercoralis* isolates from several human and dog populations in multiple countries of East Asia were examined [26]. Accordingly, two distinct lineage of *S. stercoralis* were present, referring to as type A parasites isolated both from humans and dogs, and type B parasites founding exclusively in dogs and not adapted to infect humans. All these findings suggest the possibility of zoonotic potential of *S. stercoralis* and the possibility that human *S. stercoralis* originated from dogs. Thus, dogs might be considered as a reservoir for human *S. stercoralis*. Further studies with larger samples of human and dog isolates from different geographical areas is recommended for assessing this assumption particularly in region where people have close contact with dogs.

## Conclusion

The present comparative molecular characterization of *S. stercoralis* and *Rhabditis* spp. on human-derived samples, based on *cox1* gene, indicated that this gene is a suitable marker for discrimination of *S. stercoralis* from the species of *Rhabditis* obtained in the current study. Due to the lower occurrence of *Rhabditis* spp. in human, only three isolates were available for analysis. However, this is for the first time in the world that human-dervied *Rhabditis* spp. partial sequences of *cox1* gene is compared with human isolates of *S. stercoralis* and the sequences are now available in GeneBank database. In order to find whether these sequences are representative and conserved among other pathogenic species of *Rhabditis* spp. more human and even animal retrieved isolates from different geographical areas need to be analysed, with *cox1* gene as well as other gene markers. The availability of accurate gene sequence information will be helpful in the future development and validation of discriminatory PCR-based assays, particularly ones with the low cost, rapid and simple method, for first-pass screening in research laboratories, clinical settings and epidemiological studies especially in regions with low resources.

## Data Availability

The data supporting the conclusion of the article are included in the text of the article and its additional files. The accession numbers of sequences deposited in GenBank database are provided in the relevant table of the article.

## Abbreviations

APC: Agar plate culture
*Cox1*: cytochrome c oxidase subunit 1
PCR: Polymerase Chain Reaction
qPCR: quantitative Polymerase Chain Reaction
*S. stercoralis*: *Strongyloides stercoralis*
